# Hemobilia as a Complication of Transjugular Liver Biopsy Causing Acute Pancreatitis and Obstructive Jaundice: A Case Report and Minireview

**DOI:** 10.14740/jmc5269

**Published:** 2026-02-02

**Authors:** Usama Sakhawat, Ahmed Shehadah, Behrawar Ahmad, Atif Nawaz Malik, Umar Hayat, Ali Marhaba

**Affiliations:** a Department of Gastroenterology, United Health Services Hospitals, Binghamton, NY, USA; b Department of Internal Medicine, United Health Services Hospitals, Binghamton, NY, USA; c Department of Gastroenterology, Sheikh Zayed Medical College and Hospital, Rahim Yar Khan, Pakistan; d Department of Internal Medicine, Geisinger Wyoming Valley Medical Center, Wilkes Barre, PA, USA

**Keywords:** Endoscopy, Biliary disease, Pancreatic disease

## Abstract

Liver biopsy is a critical diagnostic tool in the evaluation of certain hepatic and biliary disorders, particularly when non-invasive testing is inconclusive. It allows for definitive histopathologic assessment, guides prognostication, and informs therapeutic decision-making. Several techniques for liver biopsy are available, including percutaneous, transjugular, and surgical approaches. Among the available techniques, transjugular liver biopsy (TJLB) is considered to have a superior safety profile compared to other techniques. However, complications still occur, and hemobilia is one such rare but potentially serious event. While multiple cases of hemobilia following percutaneous liver biopsy have been reported, occurrences after TJLB are exceedingly uncommon. We present a case of hemobilia developing after a TJLB. The diagnosis can be challenging because the initial presentation is often nonspecific. In our case, the patient did not exhibit overt gastrointestinal bleeding but presented with acute pancreatitis and obstructive jaundice, which led to further evaluation and the eventual diagnosis of hemobilia. Endoscopic retrograde cholangiopancreatography (ERCP) was both diagnostic and therapeutic, resulting in complete clinical and biochemical recovery. This case highlights the importance of maintaining a high index of suspicion for hemobilia following TJLB, particularly in patients presenting with unexplained jaundice or pancreatitis even in the absence of overt gastrointestinal bleeding.

## Introduction

Transjugular liver biopsy (TJLB) is generally considered safer and associated with a lower rate of adverse events compared to the percutaneous approach. Nevertheless, complications may still occur. Hemobilia is a rare but potentially serious adverse event, with an estimated incidence of less than 0.1% among all TJLB procedures [1–3]. Other recognized causes of hemobilia include invasive hepatobiliary interventions, choledocholithiasis, and hepatobiliary malignancies.

The clinical presentation is often nonspecific, and only a minority of patients exhibit the classical Quincke’s triad of upper gastrointestinal (GI) bleeding, jaundice, and right upper quadrant pain. Diagnosis typically relies on a combination of clinical assessment, imaging studies, angiography, and endoscopic evaluation. Management is multifaceted—while most cases are self-limited, severe bleeding may necessitate transcatheter angiographic embolization or, rarely, surgical intervention. Relief of biliary obstruction often requires endoscopic retrograde cholangiopancreatography (ERCP).

We present a rare case of hemobilia following TJLB, in which the patient developed acute pancreatitis and obstructive jaundice. The diagnosis was confirmed on ERCP, which proved to be both diagnostic and therapeutic.

## Case Report

A 49-year-old male with medical history of asthma, diabetes mellitus type II, hypertension, hyperlipidemia, atrial fibrillation on apixaban, Wolff-Parkinson-White syndrome, obstructive sleep apnea, and gastro-esophageal reflux disease was found to have abnormal liver function tests (LFTs) on an annual physical exam and lab work. The patient at this time was asymptomatic with no abdominal or other systemic symptoms. LFTs - aspartate aminotransferase (AST) 71 U/L (normal < 59), alanine aminotransferase (ALT) 124 U/L (normal < 50), total bilirubin 0.5 mg/dL (normal < 1.3), and alkaline phosphatase (ALP) 37 U/L (normal 38–126). To delineate the cause of abnormal LFTs, he underwent further testing. Hepatitis B surface antigen, hepatitis B core antibody, hepatitis C Virus antibody, anti-tissue transglutaminase IgA, anti-mitochondrial antibody, and anti-nuclear antibody were negative. Ceruloplasmin levels were normal. Anti-smooth muscle antibody (ASMA) level was positive at 1:40. Since the ASMA was positive, autoimmune hepatitis was suspected.

An ultrasound (US) of the abdomen was done, which revealed a fatty liver, gallbladder sludge, and a dilated common bile duct measuring 9.6 mm. For evaluation of the dilated common bile duct, a magnetic resonance cholangiopancreatography (MRCP) was done. This showed stable dilation of the common bile duct without evidence of filling defect or stricture and hepatic steatosis.

**Figure 1:**
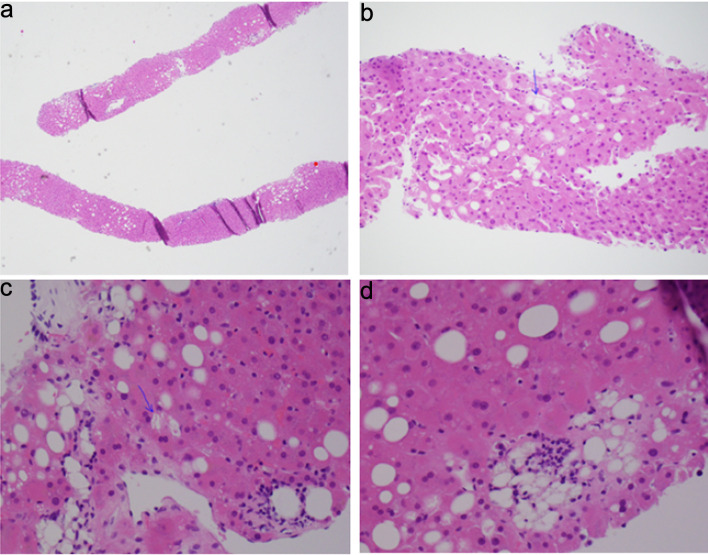
Histopathology examination of hepatic parenchyma. (a) Steatosis, involving 10–15% of hepatocytes. (b, c) Ballooning of hepatocytes (arrows). (d) Steatosis and lobular inflammation.

Since autoimmune hepatitis was suspected, the patient underwent a TJLB. Apixaban was held for 2 days prior to the procedure. The procedure was performed successfully without any intra-procedural complications. Hepatic venous pressure gradient (HVPG) was measured, which was normal, indicating no portal hypertension. Hepatic parenchymal tissue was obtained for histopathological examination. Pathology findings were consistent with fatty liver disease, possibly metabolic-associated steatohepatitis (MASH), with a non-alcoholic fatty liver disease (NAFLD) score of 3/8 (steatosis 1/3 noted in Fig. 1a, d, balloon degeneration 1/2 noted in Fig. 1b, c, and lobular inflammation 1/3 noted in Fig. 1d), mild pericellular and periportal fibrosis stage 1/4 (NASH clinical research network fibrosis staging system).

Apixaban was restarted 1 day after the procedure. Two days after the procedure, the patient presented to the emergency room with severe epigastric abdominal pain radiating to the back, along with nausea and vomiting. He denied hematemesis, hematochezia, or melena. He denied any fevers, rigors, or chills. He was hemodynamically stable with normal vitals. Lab workup was significant for abnormal LFTs—AST elevated to 113 U/L, ALT elevated to 97 U/L, total bilirubin 0.5 mg/dL, and alkaline phosphatase 48 U/L. Lipase was elevated at > 4,000 International Units/L (normal < 300). Complete blood count (CBC) revealed mild leukocytosis with a white blood cell count (WBC) of 10.9 (normal range 4–10.5 × 10^3^/µL), hemoglobin (Hb) of 13.5 g/dL (normal range 13–18 g/dL), and platelets 322 (normal range 125–425 × 10^3^/µL). Prothrombin time was slightly above normal at 13.1 s (normal 9–13 s), and partial thromboplastin time was normal at 34 s (22–37 s).

Computed tomography (CT) scan of the abdomen and pelvis showed hepatic steatosis but no acute intra-abdominal pathology. The patient was admitted to the hospital for management of acute pancreatitis.

The patient was initiated on supportive management with intravenous normal saline, analgesics, and antiemetics. The following day, there was a significant rise in LFTs. AST increased from 113 to 353 IU/L, ALT from 97 to 282 IU/L, and total bilirubin from 0.5 to 2.4 mg/dL, while alkaline phosphatase remained within normal limits. An acute decline in hemoglobin was also observed, decreasing from 13.5 to 10.6 g/dL.

At this point, the working diagnosis was acute pancreatitis of unclear etiology. On the subsequent day, LFTs continued to worsen, with AST increasing from 353 to 601 IU/L, ALT from 282 to 423 IU/L, and total bilirubin from 2.4 to 4.4 mg/dL. Alkaline phosphatase remained normal at 111 IU/L.

**Figure 2:**
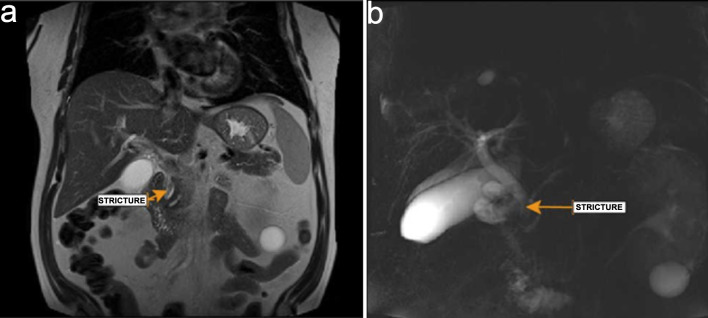
Magnetic resonance cholangiopancreatography. (a) Stricture (arrow) noted in common bile duct. (b) Dilated bile duct with distal stricture (arrow).

General surgery and gastroenterology services were consulted. In view of the recent liver biopsy and subsequent development of abnormal LFTs, obstructive jaundice, and acute anemia, hemobilia was suspected. As part of the diagnostic workup, a hepatobiliary iminodiacetic acid (HIDA) scan was performed, which demonstrated normal radiotracer uptake with no evidence of obstruction. However, MRCP revealed an elongated filling defect in the distal common bile duct with associated intrahepatic and extrahepatic ductal dilatation, findings consistent with biliary obstruction (Fig. 2a, b).

**Figure 3:**
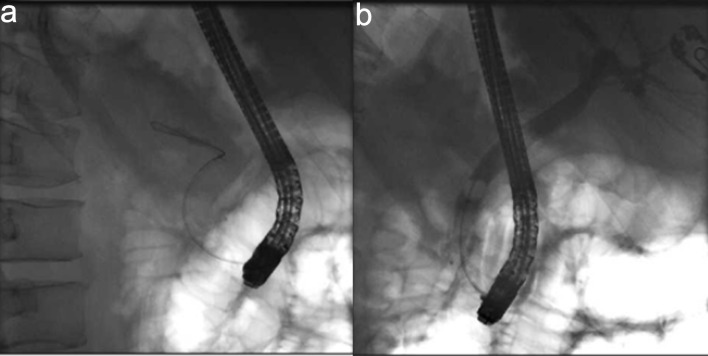
Cholangiogram. (a) Fluroscopic image revealing wire cannulation of bile duct. (b) Cholangiogram revealing a dilated biliary tree with filing defects.

**Figure 4:**
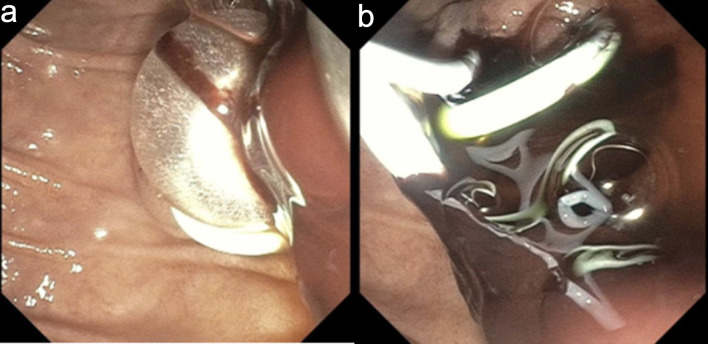
Endoscopic retrograde cholangiopancreatography. (a) Endoscopic sphincterotomy followed by balloon sweep and extraction of blood clots. (b) Plastic bile duct and pancreatic duct stents were deployed to maintain biliary drainage and to prevent post-ERCP pancreatitis, respectively.

An emergent ERCP was performed. The major papilla appeared small, and the pancreatic duct was normal. The cholangiogram demonstrated a dilated biliary tree with multiple filling defects (Fig. 3a, b). A biliary sphincterotomy was performed, and the bile ducts were swept, yielding blood clots that were subsequently removed. A prophylactic pancreatic duct stent was placed to prevent post-ERCP pancreatitis. A temporary plastic stent was placed in the common bile duct to facilitate drainage (Fig. 4a, b).

These findings confirmed hemobilia as the underlying cause of acute pancreatitis and obstructive jaundice. Following the procedure, there was complete resolution of obstructive jaundice with normalization of LFTs, and the patient recovered fully. Apixaban remained on hold at that time.

## Discussion

In this case report, we specifically describe a rare presentation of hemobilia following TJLB, complicated by obstructive jaundice and acute pancreatitis in the absence of overt GI bleeding. The diagnosis was delayed due to the lack of classic bleeding manifestations and was ultimately established by ERCP, which revealed blood clots obstructing the distal bile duct. This case highlights an uncommon but clinically important complication of a procedure generally regarded as low risk.

The focus of this discussion is the epidemiology, pathophysiology, clinical presentation, complications, and management of hemobilia.

### Epidemiology, procedural risks, and the pathophysiology

The reported incidence of significant hemobilia following all types of liver biopsy procedures is less than 0.1% [1, 2]. Theoretically, TJLB carries a lower risk of hemobilia than percutaneous biopsy. A large multicenter retrospective study by Mammen et al supported this fact, as only four cases out of 601 patients (0.67%) developed hemobilia after TJLB [3]. There have been cases of hemobilia reported following transjugular intrahepatic portosystemic shunt placement [4, 5]. Other causes of hemobilia include, but are not limited to, hepatobiliary surgery, endoscopic hepatobiliary procedures, accidental hepatic trauma, hepatocellular carcinoma, cystic artery pseudoaneurysm, severe choledocholithiasis, and spontaneous hemobilia in the setting of anticoagulation [6].

TJLB is preferred over other liver biopsy methods [7], because it minimizes the risk of free intraperitoneal hemorrhage by containing any bleeding within the vascular system and is considered more reliable in patients with coagulopathy, significant ascites, or those requiring hemodynamic measurements.

However, complications can still occur. The intrahepatic bile ducts and hepatic vasculature lie in close proximity; therefore, multiple needle passes through the liver during TJLB may injure vascular structures and lead to the formation of iatrogenic fistulae or vasculobiliary communications. These are usually arterio-biliary in nature, although veno-biliary fistulae can also occur. This mechanism underlies the pathophysiology of hemobilia and bleeding into the biliary tree [6]. In some cases, a pseudoaneurysm may develop due to procedural injury to blood vessels, which may subsequently communicate with the bile duct and form a fistula [8].

Bleeding into the biliary tree can lead to formation of clots that can cause ductal blockage, leading to obstructive jaundice [9]. Distal bile duct obstruction functionally blocks the pancreatico-biliary channel, resulting in pancreatic juice stasis. This leads to activation of pancreatic zymogens and development of acute inflammation, clinically manifesting as acute pancreatitis [10]. Serum lipase may dramatically rise up to 4,000 U/L, as reported in our case. The first ever case of acute pancreatitis due to hemobilia was documented in 1975 following percutaneous trans-hepatic cholangiogram [11].

### Clinical presentation

The signs and symptoms of hemobilia usually appear within 24 h to 7 days after the procedure [12]. The presentation can be nonspecific, often leading to a delay in diagnosis. The classic Quincke triad (jaundice, biliary colic, and upper GI bleeding) is present in only 10–25% of cases [13]. In one study, only 13.8% of patients presented with the Quincke triad [6, 14]. When hemobilia occurs, patients typically present with overt GI bleeding manifestations such as hematemesis [15] or melena. In our case, the patient did not exhibit any signs of overt GI bleeding. The diagnosis was only suspected following an acute drop in hemoglobin, without any reasonable alternative explanation for anemia other than hemobilia. There was also a significant elevation in LFTs, with a pattern suggestive of obstructive jaundice. Moreover, the presence of pancreatitis hinted at impaired drainage of the pancreatic duct due to an obstructing clot. Therefore, a high index of suspicion is required in the appropriate clinical setting to consider this diagnosis.

### Role of imaging

Cross-sectional imaging, such as CT angiography, is the first-line, non-invasive diagnostic test of choice when there is a strong suspicion of hemobilia. It can detect blood clots within the biliary system, pseudoaneurysms, and other vascular malformations [16]. However, data on the sensitivity and specificity of CT angiography in detecting hemobilia are limited. Therefore, the study may yield false-negative results, particularly in cases without overt GI bleeding.

HIDA scan has a limited role in diagnosing hemobilia. It is primarily designed to detect bile leaks and assess biliary patency. It cannot reliably distinguish between different types of fluid collections (bile, blood, pus, or serous fluid) due to their similar imaging characteristics.

The role of abdominal ultrasonography is limited, as its diagnostic utility is highly operator-dependent [6]. MRCP can also play a useful role in diagnosis; several case reports have described its confirmatory value [17]. It can identify ductal obstruction and help differentiate blood clots from stones or tumors.

Catheter-based angiography remains the gold standard for diagnosis, with a reported sensitivity of over 90%, particularly in patients with ongoing, significant overt GI bleeding manifesting as hematemesis or melena [16, 18]. Angiography can detect arterio-biliary and arterio-portal fistulae, as well as aneurysms [18]. Angiography has its limitations. Its diagnostic yield is reduced in cases of intermittent, low-volume, or self-limited bleeding, and angiographic findings may be negative in the absence of active hemorrhage at the time of the procedure. Additionally, angiography is an invasive modality.

### Management

There are two main principles of management: 1) Stabilization and control of bleeding; 2) Relief of biliary obstruction.

Initial management is conservative and includes measures necessary for hemodynamic resuscitation, correction of coagulopathy if any, holding anticoagulants if any (as in our case), correction of anemia with transfusion if necessary. In most cases, the bleeding resolves spontaneously with conservative management without the need for further intervention [14, 19].

#### Endoscopic evaluation

##### 1) Esophagogastroduodenoscopy (EGD)

EGD can play a pivotal role in diagnosis, as it allows direct visualization of the ampulla of Vater. The diagnostic yield largely depends on the rate and duration of bleeding [16]. The key endoscopic finding in hemobilia is active bleeding or blood oozing from the papilla during ongoing hemorrhage. However, when bleeding is slow or intermittent, blood may clot and cause biliary obstruction, as it does not mix with bile and instead forms separate layers due to differences in gravity and surface tension [18, 20]. In such cases, a blood clot may be visible at the ampulla—or, at times, no visible finding may be present. A side-viewing duodenoscope provides superior visualization of the papilla. Nevertheless, the absence of visible bleeding at the ampulla does not exclude the diagnosis. The diagnostic yield of endoscopy is variable; in one study, only 12% of examinations were diagnostic [19], and in a retrospective analysis, blood oozing from the ampulla of Vater was observed in only 37.5% of patients [14].

In addition, EGD can also identify and rule out other causes of upper GI bleeding, such as peptic ulcer disease or esophageal variceal bleeding.

##### 2) ERCP

ERCP has both diagnostic and therapeutic roles. Cholangiography can identify filling defects within the bile duct, while a variety of therapeutic interventions can be performed. Sphincterotomy and clot extraction relieve biliary obstruction, thereby resolving abdominal pain and jaundice [14]. In cases of uncontrolled hemobilia, a temporary metal stent may be placed for hemostatic purposes [21, 22], and balloon tamponade can also aid in achieving hemostasis. Placement of a plastic or metallic stent after clot extraction allows for continuous biliary drainage, preventing cholestasis-related complications such as cholangitis. It is important to note that bile has a natural fibrinolytic activity [19], which is impaired when bile flow is interrupted, preventing the dissolution of clots. Consequently, stagnant blood clots may persist in the bile duct due to this disruption of the bile’s fibrinolytic function.

### Impact of anticoagulation

TJLB is considered a high bleeding-risk procedure. According to Society of Interventional Radiology consensus guidelines, apixaban should be held for four doses in patients with normal renal function (CrCl ≥ 50 mL/min) prior to the procedure and resumed 24 h afterward [23]. In our case, apixaban was held for 48 h before the procedure and resumed 24 h post-procedure, in accordance with these guidelines. Although the recommended intervals for holding and resuming anticoagulation were followed, anticoagulation may still have contributed to the development or exacerbation of hemobilia in the setting of underlying vascular injury.

#### Transarterial embolization

Transarterial embolization (TAE) is a minimally invasive and effective treatment for hemobilia, with reported success rates of 80–100% [6]. Marynissen et al reported a 100% success rate with TAE without any major complications [24]. The procedure typically involves the use of microcoils or bioparticles. In our case, there was no overt GI bleeding or hemodynamic instability that would necessitate TAE. ERCP with stent placement proved adequate, and no rebleeding was observed.

### Surgery

Surgical management is considered when TAE has failed or in the presence of coexisting cholecystitis. Surgical options include ligation of the bleeding vessel and excision of a pseudoaneurysm, if present. In cases of persistent bleeding, resection of the affected liver segment may be necessary [19]. The choice of surgical approach depends on the type and severity of the injury. Nonetheless, TAE remains the first-line therapy [6].

### Conclusions

TJLB is generally preferred over the percutaneous approach due to its superior safety profile and lower risk of complications. However, adverse events may still occur, and hemobilia represents one such rare but potentially serious complication. Hemobilia following TJLB often presents atypically, requiring a high index of suspicion in the appropriate clinical context to establish the diagnosis. The diagnosis is established through a combination of clinical assessment, imaging studies, angiography, and endoscopic evaluation. Endoscopic evaluation can serve as both a diagnostic and therapeutic modality, as demonstrated in our case.

### Learning points

Hemobilia after TJLB is rare but clinically significant and often presents atypically. Despite the superior safety profile of TJLB compared with percutaneous biopsy, hemobilia can still occur and may present without overt GI bleeding. Unexplained anemia, obstructive-pattern liver enzyme abnormalities, or acute pancreatitis within days of TJLB should prompt consideration of hemobilia.

Hemobilia can cause secondary complications such as obstructive jaundice and acute pancreatitis. Intraluminal blood clots within the biliary tree may obstruct the distal bile duct, impairing biliary and pancreatic drainage. This mechanism can lead to cholestasis and pancreatitis, even in the absence of classic Quincke’s triad, underscoring the need for a high index of suspicion.

ERCP plays a crucial diagnostic and therapeutic role in hemobilia with biliary obstruction. ERCP allows direct visualization of biliary filling defects, facilitates clot extraction, and enables therapeutic interventions such as sphincterotomy and stent placement. In stable patients without ongoing hemorrhage, ERCP alone may be sufficient, obviating the need for angiographic embolization or surgical intervention.

## Data Availability

The authors declare that data supporting the findings of this study are available within the article.
